# Job satisfaction and moral distress of nurses working as physician assistants: focusing on moderating role of moral distress in effects of professional identity and work environment on job satisfaction

**DOI:** 10.1186/s12912-023-01427-1

**Published:** 2023-08-14

**Authors:** Minsub Kim, Younjae Oh, Joo Yun Lee, Eunhee Lee

**Affiliations:** 1 School of Nursing, Research Institute of Nursing Science, Hallym University, 1 Hallymdaehak-gil, Chuncheon, 24252 Gangwon-do Republic of Korea; 2 College of Nursing, Gachon University, Incheon, Korea

**Keywords:** Job satisfaction, Nurse, Physician assistant, Work environment, Professional identity, Moral Distress

## Abstract

**Background:**

Physician assistant was created in response to a shortage of physicians. However, this profession is not officially recognized in Korea. Many nurses are working as physician assistants. Their job satisfaction was low due to role conflict. Job satisfaction plays a major role in providing high quality nursing. This study aimed to investigate effects of work environment and professional identity on job satisfaction and identify the mediating role of moral distress in such effects.

**Methods:**

Participants were 112 nurses working as physician assistants. They were recruited from three General Hospitals. A questionnaire scale was used to collect data. Data were analyzed using frequency, descriptive statistics, independent t-test, one-way ANOVA, Pearson correlation, and Macro Model 4 with SPSS Statistics.

**Results:**

Job satisfaction of nurses working as physician assistants had a score of 3.08 out of 5. It showed significant associations with work environment and professional identity. Moral distress had a partial mediating role in relationships of job satisfaction with work environment and professional identity.

**Conclusion:**

Many nurses are working as physician assistants, although physician assistant as a profession is not officially recognized in Korea. Nurses working as physician assistants experience confusion about their professional identity and moral distress. For better nursing outcomes, physician assistant policy should be improved, and various strategies should be provided to improve their job satisfaction.

## Introduction

Physician assistants (PAs) are healthcare professionals who practice medicine as a part of the healthcare team under supervision of a physician. PA profession was created in the 1960s in the United States in response to a shortage of primary care physicians [1]. Recently, due to changing work hour restrictions of medical residents, PAs and nurse practitioners (NPs) have grown significantly in several countries [2, 3]. In countries where PAs formally work as a part of a healthcare team, PAs are systematically trained and formally qualified to perform a wide range of clinical duties, including diagnosing, treating illnesses, prescribing medications, and ordering diagnostic tests (1). Many studies have reported high levels of satisfaction with high-quality care by PAs, which are indistinguishable from physicians [2, 4–6].

Since healthcare system in South Korea also faced problems, such as work hour restrictions of medical residents and shortage of residents in some professional fields, PAs began to work as part of the healthcare team or replace residents in some professional fields [7, 8]. NP has also gradually emerged to address shortage of residents [3, 9]. While NPs in Korea are provided a formal education and training program, there is no accredited program for PAs in Korea [7, 8]. PAs in Korea can replace residents’ job after receiving only short apprenticeship training provided by the department where they will work without a systemic education curriculum [8]. Moreover, roles and scopes of responsibility of PAs have not been described in law or institutions where PAs work. Hence, PA in Korea is basically different from officially introduced PA by government level with proper education requirements and certification programs such as PAs in the United States, United Kingdom, and Canada [1, 5, 10–12].

Most PAs in Korea are selected and assigned among nurses because PAs not only need to possess medical knowledge and skill, but also need to have patient care competencies [7]. However, since PA in Korea has not yet been recognized as a qualified professional in the healthcare system, most nurses working as PAs are confused about their roles and identities [7, 8]. Thus, nurses working as PAs experience confusion between official qualification of nurse and their role as PA, leading to a professional identity crisis. Both professional identity crisis and role conflict can lead to negative organizational outcomes such as burnout, job stress, and job dissatisfaction [13–16]. In addition, the worse the work environment, the lower the job satisfaction [7, 16]. Especially, work environments such as workload and practice scope can vary depending on the professional field in which PA work. However, there is no common staffing standard or practice guideline [8]. Thus, PAs can experience stress and psychological discomfort, which can lead to job dissatisfaction [14, 17].

As nurses’ professional roles expand, nurses in clinical practice face various moral problem and situations. PAs and NPs also encounter many moral problems in the complex work environment. They feel frustrated and powerless, which can lead them to change or leave their jobs [18, 19]. Moral distress refers to a painful and psychologically uncomfortable feeling experienced when healthcare providers are aware of the ethically correct action to take. However, they are constrained from taking it [20]. Moral distress is a complex and challenging problem that can significantly impact the healthcare team, ranging from hindering their ability to advocate for patients to leaving their jobs or professions [20]. Moral distress of PAs and NPs can arise from a variety of causes, such as role conflict with physician, organizational constraints or policies that limit the provision of optimal care, and lack of autonomy or decision-making power in patient care [21, 22]. Hence, moral distress is correlated with both work environment and professional identity. Unfortunately, nurses working as PAs in Korea are likely to experience moral distress because the rationale for independent decision-making and their legal and ethical responsibilities are unclear [16].

Therefore, the purpose of this study was to investigate effects of work environment and professional identity on job satisfaction of nurses working as PAs and identify the mediating role of moral distress in such effects.

## Methods

### Participants and survey

This study included 112 nurses who worked as PAs from one tertiary hospital and two general hospitals with more than 500 beds in two cities of Korea. We notified these three hospitals about this study and recruited nurses working as PAs. We also recruited more nurses using snowball sampling. We included nurses working as PAs in several professional fields such as surgical part, intensive care unit, and emergency room to consider a variety of work environments. We excluded NPs even if they were working as Pas. This is because NPs in Korea differ from PAs in terms of accredited education and qualification. The survey was conducted using a questionnaire designed by researchers based on previous studies [15–22]. The questionnaire included questions about nurse characteristics, job satisfaction, and concepts associated with satisfaction.

### Variables

Individual characteristics included age, gender, education level, duration for clinical career and PA career, employment type, annual salary, and motivation for working as a PA. Work environment was measured using an instrument consisting of 28 items of six subdomains: leadership of manager, environmental supporting for work, violence in ward, appropriate supply of material and equipment, supportive environment of hospital, and satisfaction and sense of accomplishment [23]. Cronbach’s alpha of the work environment instrument using a 4-point Likert scale was 0.91 in this study.

Professional identity was measured using the instrument by Kim [24], which has four subdomains: belief in autonomy (4 items), belief in service (3 items), vocation for a job (6 items), and professional organizational activities as a criterion (4 items). Professional identity was regarded as a high professional identity (close to 85). Cronbach’s alpha of the professional identity instrument using a 5-point Likert scale was 0.81 in this study.

Moral sensibility was included in the model as a covariate. It was measured using Korean K-MSQ tool [25], which has five subdomain (27 items): ordinate scope, patient-centric nursing, professional liability, conflict, moral sense, and virtue. The higher the total score, the higher the moral sensitivity. Cronbach’s alpha of the moral sensitivity tool using a 7-point Likert scale was 0.85 in this study.

Job satisfaction was measured using an instrument consisting of 20 items [26] in seven subdomains: reward (2 items), professional position (3 items), administration (3 items), autonomy (3 items), demands of the task (4 items), interaction (3 items), and relationship with doctor or staff from related departments (2 items). The higher the total score, the higher the job satisfaction. Cronbach’s alpha of the job satisfaction instrument using a 5-point Likert scale was 0.75 in this study.

Moral distress was measured using the instrument consisting of 21 items [27] in five subdomains: treatment for life-prolonging, nursing practice, context factors, limitations on claiming on ethical issues, and doctor’s practice. Moral distress was calculated by multiplying the score of each questions’ frequency and strength of moral distress in each question. Since the frequency was measured with 0 point (never) to 4 point (more than 4 times) and the strength was measured with 0 point (never) to 4 point (very strong), the total score ranged from 0 to 336. The higher the total score, the stronger the moral distress. Cronbach’s alpha of the moral stress instrument was 0.92 in this study.

### Analysis

Characteristics of participants were summarized using descriptive statistics. Participants’ work environment, professional identity, job satisfaction, moral sensitivity, and moral distress are reported as mean and standard deviation. Independent t-test and one-way ANOVA) were used to assess the difference in job satisfaction according to participants’ characteristics. Relations among work environment, professional identity, job satisfaction, moral sensitivity, and moral distress were analyzed using Pearson’s correlation coefficient. SPSS PROCESS Macro by Hayes (2013) was used for analyzing the mediating effect [28]. Macro Model 4 of Hayes (2013) was used to understand the mediational effect of moral distress on paths in which work environment and professional identity affected job satisfaction [28]. Bootstrapping can accurately show indirect effects even when the sample size is small because it does not assume normality of sampling distribution. Therefore, this study conducted analysis using sex, education, and moral sensibility as control variables, extrapolating 10,000 bootstrapping samples and applying a 95% confidence interval. Path A was the direct effect of work environment or professional identity on moral distress. Path B was the direct effect of moral distress on job satisfaction. Lastly, Path C showed a direct effect path of professional identity on job satisfaction. The multiplication product of Path A and Path B was shown to demonstrate an indirect effect.

### Ethical consideration

This study received ethical approval from GangNeung Asan Hospital (No. GNAH 2020-07-004-001). Written informed consent for the survey was obtained from all participants. Permission to use the instrument was granted by its developers.

## Results

### Baseline characteristics of physician assistant nurses

Table 1 presents characteristics of 112 physician assistant nurses. Most (67.0%) nurses working as PA were women. Their mean age was 31.8 years. Nurses with a bachelor’s degree accounted for 65.2%, followed by nurses with an associate degree (22.3%) and those who went to a graduate school (12.5%). Average career was 8.6 years in total and 3.6 years for PA. The majority of nurse working as PA was employed in a permanent job (92.9%). Their annual income was over 40 million won. 43% of nurses decided to work as PA for avoiding rotating shift, followed by manpower arrangement (25.0%), advanced practice (21.4%), professional self-development (8.0%), and others (1.8%).

**Table 1:** Job satisfaction according to characteristics of participants (N = 112)

Characteristics	Categories			n (%)		Job satisfaction	
						M ± SD	t/F(*p*)
Gender	Male			37(33.0)		3.17 ± 0.43	1.579 (0.117)
	Female			75(67.0)		3.03 ± 0.45	
Age(year)	20 ~ 29			48(42.9)		3.13 ± 0.45	1.021 (0.364)
	30 ~ 39			50(44.6)		3.01 ± 0.44	
	40 ~ 49			14(12.5)		3.14 ± 0.49	
Education	Associate degree			25(22.3)		2.91 ± 0.37	2.307 (0.104)
	Bachelor			73(65.2)		3.13 ± 0.46	
	Graduate school			14(12.5)		3.11 ± 0.47	
Clinical career	Within 5 years			40(35.8)		3.17 ± 0.46	1.445 (0.240)
	5–10 years			37(33.0)		3.04 ± 0.40	
	Over 10 years			35(31.2)		3.01 ± 0.47	
PA career	Within 1 year			30(26.8)		2.94 ± 0.46	1.595 (0.195)
	1–3 years			39(34.8)		3.10 ± 0.43	
	3–5 years			18(16.1)		3.09 ± 0.44	
	Over 5 years			25(22.3)		3.20 ± 0.44	
Employment type	Permanent job			104(92.9)		3.09 ± 0.45	1.165 (0.247)
	Temporary job			8(7.1)		2.90 ± 0.35	
Annual income	Within 40 million won			30(26.8)		3.04 ± 0.41	− 0.555 (0.580)
	Over 40 million won			82(73.2)		3.09 ± 0.46	
Working motivation	Avoiding rotating shift			49(43.8)		3.04 ± 0.45	1.344 (0.259)
	Manpower arrangement			28(25.0)		3.03 ± 0.43	
	Advanced practice			24(21.4)		3.23 ± 0.38	
	Self-development			9(8.0)		3.13 ± 0.59	
	Others			2(1.8)		2.68 ± 0.46	

### Work environment, professional identity, moral sensibility, job satisfaction, moral distress levels, and their correlations

The average job satisfaction score was 3.08 out of 5. Regarding subdomains of job satisfaction, the average work environment score was 2.58 out of 4. Regarding subdomains of job satisfaction, interaction had the highest score (3.86) and reward had the lowest score (2.39). The work environment score was 2.58 out of 4. Satisfaction and sense of accomplishment had the highest score (2.84) while supportive environment of hospital had the lowest score (2.37). Average professional identity score was 56.30, ranging from 37 to 76. The average score of four subdomains (belief in autonomy, belief in service, vocation for a job, and professional organizational activities) as criteria showed similar levels (3.28–3.33). Moral sensitivity score in this study was 4.92 out of 7. Moral distress score in this study was 88.02, ranging from 0 to 279 (Table 2).

**Table 2:** Levels of work environment, professional identity, moral sensitivity, job satisfaction, and moral distress of physician assistants

Variables	Mean ± SD	[Min-Max]
Job satisfaction	3.08 ± 0.45	[2–4]
Work environment	2.58 ± 0.42	[1–3]
Professional identity	56.30 ± 8.62	[37–76]
Moral distress	88.02 ± 64.71	[0-279]
Moral sensitivity	4.92 ± 0.65	[2–7]

In terms of correlations among the five variables, job satisfaction was significantly correlated with work environment (r = 0.65, *p* < 0.01), professional identity (r = 0.61, *p* < 0.01), and moral distress (r = -0.37, *p* < 0.01). However, moral sensitivity was not significantly correlated with the other four variables. Based on correlation analysis between mediate variable and predictors, moral distress only showed a significant correlation with work environment (r = -0.32, *p* < 0.01) (Table 3).

**Table 3:** Correlations among work environment, professional identity, moral sensitivity, job satisfaction, and moral distress

	Work environment	Professional identity	Moral sensitivity	Moral distress	Job Satisfaction
Work environment	1				
Professional Identity	0.58 (< 0.01)	1			
Moral Sensitivity	0.06 (0.56)	0.14 (0.14)	1		
Moral Distress	− 0.32 (< 0.01)	− 0.19 (0.06)	0.16 (0.11)		
Job Satisfaction	0.65 (< 0.01)	0.61 (< 0.01)	0.07 (0.44)	− 0.37 (< 0.01)	1

### Mediated effect of moral distress in the relation between work environment and professional identity on job satisfaction

**Fig. 1:**
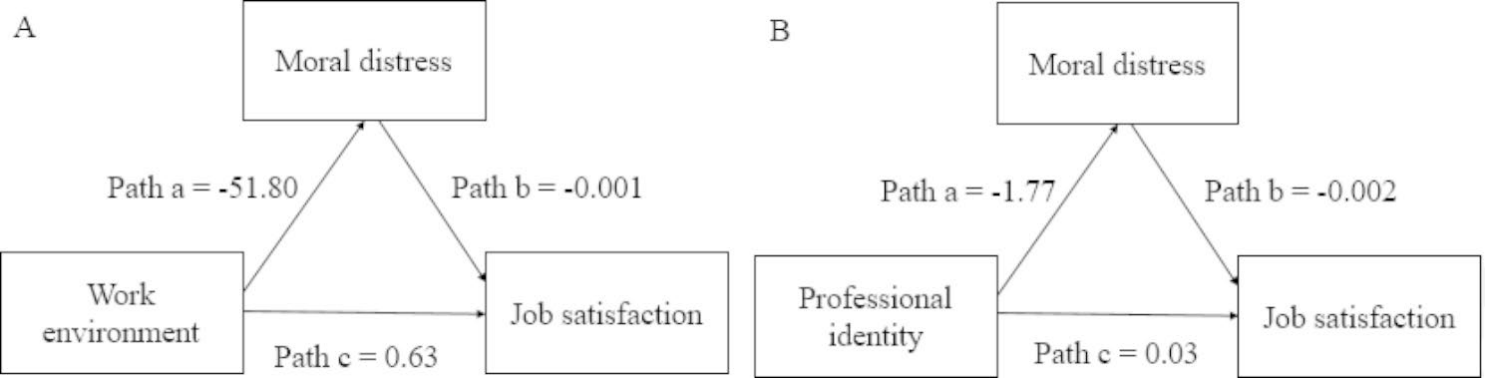
Mediation effect of the relationship between professional identity and work environment on job satisfaction through moral distress

Analysis was conducted using Hayes’ PROCESS Macro Model 4. Results are shown in Table 4; Fig. 1. When sex, education, and moral sensibility were controlled for, work environment had a direct negative effect on moral distress (b = -51.80, *p* < 0.01), moral distress had a direct negative effect on job satisfaction (b = -0.001, *p* = 0.02), and work environment had a direct positive effect on job satisfaction (b = 0.63, *p* < 0.01). The total effect, which was the sum of direct effects and indirect effects, was positive (b = 0.70, *p* < 0.01). When sex, education, and moral sensibility were controlled for, professional identity had a direct negative effect on moral distress (b = -1.77, *p* = 0.02), moral distress had a direct negative effect on job satisfaction (b = -0.002, *p* < 0.01), and professional identity had a direct positive effect on job satisfaction (b = 0.03, *p* < 0.01). The total effect was positive (b = 0.03 and *p* < 0.01).

**Table 4.1:** Mediating effect of moral distress on the relationship between work environment and job satisfaction

Variables	b	SD	t	p	LLCI	ULCI	
					95%CI	95%CI	
(Constant)	111.75	62.75	1.78	0.08	-12.84	236.33	
X1 → M (Path a)	-51.80	14.43	-3.59	< 0.01	-80.46	-23.15	
Covariate (Moral sensitivity)	18.61	9.44	1.98	0.05	-0.13	37.35	
Covariate (Female)	29.23	13.52	2.16	0.03	2.39	56.08	
Covariate (Associate degree)	-15.50	20.24	-0.77	0.45	-55.70	24.69	
Covariate (bachelor’s degree)	1.26	18.42	0.07	0.95	-35.32	37.83	
Model fit	R^2^ = 0.18, F = 4.15, p < 0.01						
(Constant)	1.54	0.36	4.32	< 0.01	0.83	2.25	
X1 → Y (Path c)	0.63	0.09	7.34	< 0.01	0.46	0.80	
M → Y (Path b)	-0.001	0.001	-2.35	0.02	-0.002	-0.0002	
Covariate (Moral sensitivity)	0.02	0.05	0.45	0.66	-0.08	0.13	
Covariate (Female)	-0.11	0.08	-1.37	0.17	-0.26	0.04	
Covariate (Associate degree)	-0.17	0.11	-1.52	0.13	-0.40	0.05	
Covariate (bachelor’s degree)	0.05	0.10	0.45	0.66	-0.16	0.25	
Model fit	R^2^ = 0.49, F = 15.08, p < 0.01						
Total effect							
(Constant)	1.39	0.36	3.87	< 0.01	0.68	2.10	
X1 → Y	0.70	0.08	8.49	< 0.01	0.54	0.87	
Covariate (Moral sensitivity)	-0.001	0.05	-0.02	0.98	-0.11	0.11	
Covariate (Female)	-0.15	0.08	-1.88	0.06	-0.30	0.01	
Covariate (Associate degree)	-0.15	0.12	-1.31	0.19	-0.38	0.08	
Covariate (bachelor’s degree)	0.04	0.11	0.42	0.67	-0.17	0.25	
Model fit	R^2^ = 0.46, F = 16.22, p < 0.01						

b: regression coefficient; CI: confidence interval; X1: work environment; M: moral distress;

Y: job satisfaction

**Table 4.2:** Mediating effect of moral distress on the relationship between professional identity and job satisfaction

Variables	b	SD	t	p	LLCI	ULCI	
					95%CI	95%CI	
(Constant)	83.28	69.34	1.20	0.23	-54.39	220.95	
X2 → M (Path a)	-1.77	0.77	-2.30	0.02	-3.31	-0.24	
Covariate (Moral sensitivity)	19.48	9.82	1.98	0.05	-0.02	38.98	
Covariate (Female)	25.17	14.03	1.79	0.08	-2.67	53.02	
Covariate (Associate degree)	-25.85	22.03	-1.17	0.24	-69.59	17.90	
Covariate (bachelor’s degree)	-5.08	19.76	-0.26	0.80	-44.32	34.16	
Model fit	R^2^ = 0.12, F = 2.51, p = 0.035						
(Constant)	1.35	0.38	3.54	< 0.01	0.59	2.11	
X2 → Y (Path c)	0.03	0.004	7.07	< 0.01	0.02	0.04	
M → Y (Path b)	-0.002	0.001	-3.39	< 0.01	-0.003	-0.001	
Covariate (Moral sensitivity)	0.01	0.05	0.27	0.79	-0.09	0.12	
Covariate (Female)	-0.03	0.08	-0.44	0.66	-0.19	0.12	
Covariate (Associate degree)	0.03	0.12	0.23	0.82	-0.21	0.27	
Covariate (bachelor’s degree)	0.19	0.11	1.76	0.08	-0.02	0.40	
Model fit	R^2^ = 0.48, F = 14.30, p < 0.001						
Total effect							
(Constant)	1.19	0.40	2.99	< 0.01	0.40	1.99	
X2 → Y	0.03	0.005	7.66	< 0.01	0.03	0.04	
Covariate (Moral sensitivity)	-0.02	0.06	-0.40	0.69	-0.13	0.09	
Covariate (Female)	-0.08	0.08	-1.02	0.31	-0.24	0.08	
Covariate (Associate degree)	0.08	0.13	0.61	0.54	-0.17	0.33	
Covariate (bachelor’s degree)	0.20	0.11	1.75	0.08	-0.03	0.43	
Model fit	R^2^ = 0.42, F = 13.37, p < 0.001						

b: regression coefficient; CI: confidence interval; X2: professional identity; M: moral distress;

Y: job satisfaction

Table 5 shows results of analysis of indirect effects to verify the mediational effect of moral distress on the relationship of work environment and professional identity with job satisfaction. In the relationship between work environment and job satisfaction, the size of the mediational effect of moral distress was 0.0702 (95% CI: 0.0024–0.1539), which was statistically significant since bootstrap CI did not include 0. In the relationship between professional identity and job satisfaction, the size of the mediational effect of moral distress was 0.0034 (95% CI: 0.0003–0.0072), which was statistically significant since bootstrap CI did not include 0.

**Table 5:** Indirect effects

Variables	Effect	Boost SE	Boot LLCI	Boot ULCI
			95% CI	95% CI
Work environment	0.0702	0.0390	0.0024	0.1539
Professional identity	0.0034	0.0018	0.0003	0.0072

## Discussion

This study investigated the impact of work environment and professional identity on job satisfaction of nurses working as PAs and identified the mediating effect of moral distress in this relationship. Job satisfaction level of nurses working as PAs was moderate (3.08 out of 5), similar to other studies targeting nurses working as PAs in Korea [16]. However, it was lower than PAs and NPs [2, 4, 5, 17, 29, 30]. This gap in job satisfaction between PAs in Western countries and Korea might be attributed to differences in PA model such as practice scope, education requirements, and legal and institutional recognition. PAs in Korea have not yet been recognized as official professionals in the healthcare system yet. Thus, there is no accredited training program or practice guideline for PAs [8]. However, many staff are performing PA job in actual clinical practice, although PA profession is not officially recognized in Korea. In addition, most staff are nurses because they are qualified in holistic patient care with medical knowledge and skills. Hence, many nurses working as PAs who have to perform officially unrecognized jobs without training struggle with professional identity and role conflict, leading to job dissatisfaction [13, 16]. Since job dissatisfaction ultimately leads to turnover [31, 32] and low-quality care [33], job satisfaction of nurses working as PA should be managed.

This study showed that work environment and professional identity were factors influencing job satisfaction of nurses working as PAs, consistent with other studies [15, 16, 32, 34, 35]. The more nurses working as PA perceived their work environment to be poor, the more they were dissatisfied with their work. In addition, moral distress mediated the relationship between work environment and job satisfaction. Since work environment includes several supportive systems of hospital such as material supply and environmental supporting, nurses in poor work environment are likely to experience moral distress. Moral distress referring to a psychological discomfort arises when a person is aware of the right course to take but is prevented from acting on it by institutional constraints [20]. Many previous studies have found that moral distress is caused by institutional and context factors [20, 36–38]. For nurses working as PA in South Korea, the unclearly defined PA work and the organizational atmosphere in which PAs are not officially recognized might have led them to experience a lot of moral distress while performing their job. In the present study, not only work environment, but also professional identity affected moral distress.

Professional identity refers to having the authority to make decisions and freedom to act in accordance with one’s professional knowledge base. Many previous studies have indicated a high level of moral distress in nurses who encounter patients primarily with little independent authority [21, 39]. In Korea, where PA job is not officially recognized and PA has no independent authority over the job, nurses working as PAs encounter many situations in which they have to perform tasks that exceed their responsibilities as nurses. For example, nurses working as PAs in Korea can prescribe and perform some medical treatment outside the RN scope of practice [7]. Moreover, as their practices are not even legally protected, legal and ethical issues in their practices are sometimes raised [8]. Hence, nurses working as PAs are experiencing a role conflict between their legal status and the work they perform. They are asked to perform practices requiring high authority. This situation ultimately leads to job dissatisfaction through a high moral distress [16]. To increase job satisfaction of nurse working as PA, it is preferentially necessary to establish their job identity by clarifying their roles and scopes of practice. When professional identity is strengthened through legal and organizational changes, moral distress experienced by nurses working as PAs is expected to decrease and job dissatisfaction due to this will also decrease.

In our study, the moral distress level of nurses working as PA was 88.02. Although it was difficult to compare this study to other studies due to large variations in moral distress depending on working place and country, the level of moral distress in this study was lower than that of nurses working at ICU [40]. Since previous studies have shown that moral distress is influenced by frequency of exposure to ethical situations [20, 36], moral distress of nurses working as PAs is expected to be lower than that of nurses working as critical care unit who frequently encounter ethical situations such as end-of-life nursing and end-of-life patient nursing. However, the level of moral distress of nurses working as PA in this study was significantly higher than that of most nurses working in general wards [38, 41, 42]. The high moral distress in this study might be attributed to limited decision authority of nurses working as PA and their poor work environment. Unlike other countries where PA is officially recognized as a job and produced through an accredited program [1, 43], many nurses are performing PA duties as needed by healthcare institutions without any accredited training [8]. Thus, many nurses working as PAs have experienced high moral distress, which can lead to job dissatisfaction. In this study, it was also found that moral distress affected job satisfaction, consistent with other studies targeting PAs and nurses [16, 44]. In addition to job satisfaction, many studies have revealed that moral distress could directly affect burnout of healthcare workers [30, 45]. Thus, healthcare professionals’ high moral distress should be managed to improve organizational outcomes.

Many countries have introduced PAs and NPs to solve the shortage problem of physicians. They formally become part of the qualified healthcare team (6, 9). Based on qualification through accredited education, PAs and NPs can provide high-quality of care to patients [4, 5, 17]. Their performance has been reported by many previous studies [2, 5, 17]. Unlike countries where PA is officially introduced by the government with proper education, South Korea has many nurses working as PAs without any official recognition or systematic training. To have better outcomes for patients and nurses, we need to discuss the PA system in Korea. Hospitals and nurse managers should establish various strategies to increase job satisfaction of nurses who current perform the role of PA until systems such as practice guideline and training program are established at the government level.

This study has several limitations. Results on the influence derived from this study could not be generalized because this study included nurses working in hospitals with similar characteristics to investigate relationships among work environment, professional identity, moral distress, and job satisfaction. Further research comparing job satisfaction and moral distress according to hospital size and scope of practice is needed. Lastly, caution is needed when generalizing results of this study to other areas because of its limited sample size. A large-scale study is needed to draw definite conclusions.

## Conclusion

Nurses working as PA in Korea experience moral distress. They are dissatisfied with their job due to moral distress. Work environment and professional identity are significant factors influencing job satisfaction of nurses working as PAs. Moral distress mediated these relationships. Since the PA profession is not officially recognized in Korea, many nurses are working as PAs without receiving an accredited education. Nurses working as PAs are performing duties outside of nurses’ job, even though they are nurses. Thus, they experience confusion about their professional identity and moral distress. The Korea government should improve PA policy for better patient and nurse outcomes. Hospitals should provide various strategies to improve their job satisfaction.

## Data Availability

The datasets used and/or analyzed during the current study are available from the corresponding author upon reasonable request.
